# Recurrent otitis media and behaviour problems in middle childhood: A longitudinal cohort study

**DOI:** 10.1111/jpc.16518

**Published:** 2023-11-14

**Authors:** Ali AH Altamimi, Monique Robinson, Eman MA Alenezi, Tamara Veselinović, Robyn SM Choi, Christopher G Brennan‐Jones

**Affiliations:** ^1^ Telethon Kids Institute The University of Western Australia Perth Western Australia Australia; ^2^ School of Medicine The University of Western Australia Perth Western Australia Australia; ^3^ Faculty of Life Sciences Kuwait University Kuwait City Kuwait; ^4^ Faculty of Allied Health Sciences Kuwait University Kuwait City Kuwait; ^5^ School of Human Sciences The University of Western Australia Perth Western Australia Australia; ^6^ Audiology Department Perth Children's Hospital Perth Western Australia Australia; ^7^ School of Allied Health Faculty of Health Sciences, Curtin University Perth Western Australia Australia

**Keywords:** attention, behavioural development, developmental outcome, otitis media

## Abstract

**Aim:**

To investigate the long‐term effects of early‐life recurrent otitis media (OM) and subsequent behavioural problems in children at the age of 10 years.

**Methods:**

Data from the Raine Study, a longitudinal pregnancy cohort, were used to categorise children into those with three or more episodes of OM (rOM group) and those without a history of recurrent OM in the first 3 years of life (reference group). The parent report Strengths and Difficulties Questionnaire was used to assess child behaviour at the age of 10 years. Parental questionnaires were used to report past and present diagnoses of various mental health and developmental conditions, including attention, anxiety, depression, learning, and speech‐language problems. Multiple linear and logistic models were used to analyse the data and were adjusted for a fixed set of key confounding variables.

**Results:**

The linear regression analysis revealed significant, independent associations between a history of recurrent OM and higher Strengths and Difficulties Questionnaire scores, including total, internalising, externalising, emotional, attention/hyperactivity and peer problems subscales. Logistic regression analyses revealed an independent increased likelihood for children in the rOM group to have a diagnosis of attention, anxiety, learning and speech‐language problems.

**Conclusion:**

Children at 10 years of age with an early history of recurrent OM are more likely to exhibit attentional and behavioural problems when compared to children without a history of recurrent OM. These findings highlight the association between early‐life recurrent OM and later behavioural problems that may require professional allied health‐care interventions.

## What is already known on this topic

1. Recurrent otitis media (OM) is common in early childhood.
2. Children with recurrent otitis media can experience long‐term conductive hearing loss.
3. Previous studies have found evidence linking OM‐related hearing loss with long‐term developmental problems including behaviour, language, and mental health.

## What this paper adds

1. Further evidence suggests an association between early‐life recurrent OM and behaviour problems at the age of 10 years.
2. Parental reports indicate children with an early‐life recurrent OM are at an increased risk of having long‐term independent diagnoses of attention, language, anxiety and learning problems.

Middle ear inflammation, also known as otitis media (OM), is a term that refers to a spectrum of inflammatory conditions and is one of the most common conditions in early childhood.^1^ A differentiator between acute OM (AOM) and OM with effusion (OME) is the presence of signs of active infection, such as pain and fever, which are observed in the former and typically absent in the latter. However, both forms typically result in effusion in the middle ear, leading to various degrees of conductive hearing loss.^2^ These symptoms can be prolonged if OM persists or recurs, and may expose children to an increased risk of developmental problems. For example, previous studies that explored the relationship between a history of recurrent and/or persistent OM and behavioural problems reported increased internalising (i.e., inner‐directed) and externalising (i.e., outer‐directed) behavioural problems with emotional, social, attention, and hyperactivity being amongst the most commonly reported problems.^3^, ^4^, ^5^, ^6^, ^7^, ^8^ These problems persisted into the early adolescent years, suggesting a potential long‐term impact even when any OM‐related symptoms have presumably resolved. However, the limitations and methodological designs in previous studies do not provide sufficient evidence to support the relationship between OM and behavioural problems, the extent of these problems, and whether they are of clinical significance. In other words, behavioural screening tools are designed to provide information regarding present behavioural status but do not confirm behavioural problem diagnoses. Therefore, it is crucial to understand the nature of these associations, if any, as behavioural problems in early childhood may interfere with a child's academic achievement.^9^ This study aimed to investigate: (i) whether an early history of recurrent OM can result in behavioural problems in children at 10 years of age; and (ii) the likelihood of these children receiving a clinical diagnosis for mental health or a developmental disorder from a health professional.

## Methods

### The Raine Study participants

A total of 2900 pregnant women (Gen 1) at 16–20 gestation weeks were recruited in the Raine Study between May 1989 and November 1989 at King Edward Memorial Hospital (KEMH) and other private clinics in Perth, Western Australia (WA). Gen 1 participants were required to speak English proficiently to understand the implications of participation, had to expect to deliver their child at KEMH and continue residency in WA for follow‐up purposes. A total of 2868 children in Gen 2 were comprehensively followed up and assessed for various health and development aspects, including OM and behavioural development. The follow‐up process involved surveys that were completed by Gen 1 participants prior to each follow‐up visit. These were then verified on the day of the follow‐up visit of Gen 2 participants by a research nurse or assistant, who also performed additional assessments. The Raine Study design has been detailed elsewhere.^10^

### Ethical considerations

The human ethics committee at KEMH and Princess Margaret Hospital approved participation and follow‐up for the Raine Study. Written informed consent was obtained from all Gen 1 participants at the time of recruitment. Written informed consent was also obtained at each follow‐up phase. All Gen 2 participants further consented when they were 18 years of age. The release of data for the current study was approved by the Raine Study Scientific Review Committee.

### The present study

#### Identification of recurrent OM

To determine the history of OM in children in their first 3 years of life, caregivers were asked, ‘has your child ever had middle ear infections (i.e., OM) in his/her life?’, ‘if yes, how many times?’. On this basis, two groups were formed. Children reported to have three or more episodes of OM (range: 3–8) in the first 3 years of life were classified with recurrent OM (rOM group). Children without a history of OM and those with two or fewer reported episodes of OM were considered the reference group.

#### Outcome measures

The parent report Strengths and Difficulties Questionnaire (SDQ) and a parental questionnaire (PQ) were administered at the 10‐year follow‐up. The SDQ is a widely used screening tool to assess behavioural strengths and difficulties in children and adolescents aged 2–17 years.^11^ It consists of five subscales that measure emotional and behavioural functioning and include: emotional symptoms, conduct problems, hyperactivity‐inattention, and peer problems, in addition to the prosocial subscale that measures personal strengths. The summing of all subscales, except for the prosocial subscale, generates a total SDQ score. Further, internalising scores can be generated by the sum of the emotional and peer subscales, while externalising scores involve the sum of the conduct and hyperactivity‐inattention subscales. Scoring of the SDQ is based on a 3‐point Likert scale where ‘*not true*’ equals 0, ‘*somewhat true*’ equals 1 and ‘*certainly true*’ equals 2. Higher scores are indicative of poorer behavioural outcomes. The SDQ is available in self‐report, teacher, and parent report versions (www.sdqinfo.org). The current study also used the PQ to obtain information regarding current and previous diagnoses of various health and developmental problems. Caregivers were asked, ‘Does your child have now, or has your child had in the past, any of the following health professional diagnosed medical conditions or health problems?’. These included anxiety, attention, depression, speech and language, and learning problems, amongst other health and medical conditions. This question was answered: (1) no, (2) yes in the past, (3) yes‐now, and (4) yes‐now and in the past. This study only considered conditions that were persistent at the age of 10 years by dichotomising the answers to numbers 1 and 2 as ‘no’ and the answers to numbers 3 and 4 as ‘yes’.

### Statistical analysis

Descriptive statistics were conducted to summarise the characteristics of the study population and the frequency of each categorical variable for the SDQ and the PQ. Multiple linear regression models were used for cross‐sectional comparisons of both groups and to predict the change in each SDQ subscale and the total, internalising, and externalising scales. The predictor variable used was a history of recurrent OM in the first 3 years of life. A logistic regression was performed to ascertain the effects of recurrent OM on the likelihood of participants being diagnosed with any of the following mental health and developmental problems: anxiety, depression, attention, learning and speech‐language. All regression analyses were adjusted for key confounding variables previously identified in the literature, including sex, ethnicity, exposure to passive smoking, family income, day care attendance, premature birth and birthweight.^12^ For all analyses, a *P* ≤ 0.05 was considered statistically significant. Data were analysed using IBM SPSS Statistics software version (28.0.1.1), Armonk, NY, USA.

### Results

**Fig. 1:**
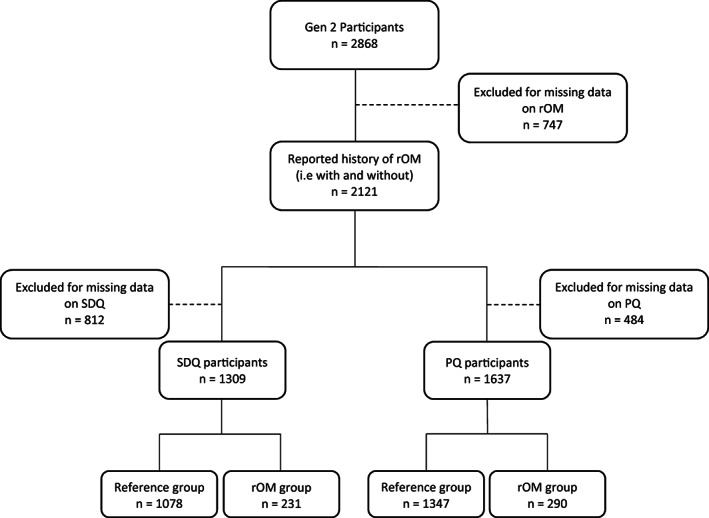
Flowchart of participants who were included in the analysis of the Strengths and Difficulties Questionnaire (SDQ) and the parental questionnaire (PQ).

A total of 2121 participants had data available regarding the history of OM in the first 3 years of life. Of those participants, 1755 (82.7%) participants were not reported to have recurrent OM, while 366 (17.3%) were reported to have recurrent OM. A total of 1309 out of 2121 (61.7%) had complete SDQ data and information on confounding variables recorded, with 1078 (83.4%) in the reference group and 231 (17.6%) in the rOM group (Fig. 1). Further, a total of 1637 out of 2121 (77.2%) participants had complete information on the PQ and confounding variables, with 1347 participants (82.3%) in the reference group and 290 participants (17.7%) in the rOM group. A summary of the sociodemographic variables of the participants is listed in Table 1.

**Table 1:** Demographic, obstetric and environmental characteristics of study participants Included in this study.

	SDQ		PQ	
	rOM group (*n* = 231), % (*n*)	Reference group (*n* = 1078), % (*n*)	rOM group (*n* = 290), % (*n*)	Reference group (*n* = 1347), % (*n*)
Sex				
Males	52.8 (122)	52.6 (567)	54.8 (159)	51.7 (696)
Females	47.2 (109)	47.4 (511)	45.2 (131)	48.3 (651)
Ethnicity				
Caucasian	96.1 (222)	89.1 (960)	96.9 (281)	89.1 (1200)
Other	3.9 (9)	10.9 (118)	3.1 (9)	10.9 (147)
Exposure to passive smoking				
Yes	40.7 (94)	41.6 (448)	40.3 (117)	42.7 (575)
No	59.3 (137)	58.4 (630)	59.7 (173)	57.3 (772)
Annual income <$27 000				
Yes	35.1 (81)	37.6 (405)	36.9 (107)	39.3 (530)
No	64.9 (150)	62.4 (673)	63.1 (183)	60.7 (817)
Birthweight <2500 g				
Yes	5.2 (12)	6.7 (72)	4.8 (14)	6.7 (90)
No	94.8 (219)	93.3 (1006)	95.2 (276)	93.3 (1257)
Day care attendance				
Yes	62.8 (145)	47.7 (514)	64.8 (188)	48.8 (657)
No	37.2 (86)	52.3 (564)	35.2 (102)	51.2 (690)
Pre‐term birth <37 weeks				
Yes	5.6 (13)	6.8 (73)	5.5 (16)	7.1 (96)
No	96.4 (218)	93.2 (1005)	94.5 (274)	93.1 (1251)

PQ, parental questionnaire; rOM, recurrent otitis media; SDQ, Strengths and Difficulties Questionnaire.

The multiple linear regression analysis revealed a statistically significant association between recurrent OM in the first 3 years of life and the total, internalising, externalising, hyperactivity, emotional, and peer subscales after adjusting for all predictor variables. This indicates that children in the rOM group demonstrated greater difficulties on these subscales when compared to the reference group. In contrast, there were no significant differences between the groups on the conduct and prosocial subscales (Table 2).

**Table 2:** Results of multiple linear regression models comparing the effects of recurrent OM on SDQ scores at age 10 years after adjusting for all predictor variables.

	Unstandardised β	SE	*P*‐value	95% CI
Total	1.10	0.39	0.005**	(0.33, 1.86)
Internalising	0.54	0.21	0.01**	(0.13, 0.95)
Externalising	0.55	0.24	0.022*	(0.08, 1.02)
Hyperactivity	0.40	0.16	0.028*	(0.04, 0.68)
Emotional	0.28	0.13	0.037*	(0.02, 0.54)
Peer	0.27	0.12	0.023*	(0.04, 0.5)
Conduct	0.12	0.12	0.10	(−0.04, 0.42)
Prosocial	−0.08	0.12	0.50	(−0.31, 0.12)

* *P* ≤ 0.05;

** *P* ≤ 0.01.

β, unstandardised coefficients; CI, confidence interval; OM, otitis media; SDQ, Strengths and Difficulties Questionnaire; SE, standard error.

The logistic regression analysis revealed independent increased odds for the rOM group to have a diagnosis in all of the health and developmental conditions listed in Table 3 whilst adjusting for all confounding variables. However, this was only significant for attention, anxiety, learning, and speech and/or language problems.

**Table 3:** Results of binary logistic regression models assessing the effects of recurrent otitis media on the likelihood of receiving a diagnosis of a mental health or developmental problem.

Diagnoses	Unstandardised β	SE	*P*‐value	OR	95% CI
Attention	0.56	0.20	0.008**	1.74	(1.16, 2.62)
Anxiety	0.77	0.35	0.029*	2.16	(1.08, 4.32)
Depression	0.85	0.49	0.082	2.33	(0.89, 6.04)
Speech	0.79	0.23	<0.001***	2.20	(1.40, 3.45)
Learning	0.44	0.20	0.035*	1.55	(1.03, 2.33)

*** *P* ≤ 0.001;

** *P* ≤ 0.01;

* *P* ≤ 0.05.

β, unstandardised coefficients; CI, confidence interval; OR, odds ratio; SE, standard error.

## Discussion

This study investigated whether early‐life recurrent OM was associated with behavioural problems at 10 years of age and whether recurrent OM was associated with an increased likelihood of health professional diagnoses for mental health or developmental problems. The results revealed that children with early‐life recurrent OM exhibited poorer internalising and externalising behaviour scores than the reference group, as measured by the SDQ. Additionally, these children were more likely to be diagnosed with anxiety, learning, speech and language, and attention‐related problems that were present before and at 10 years of age. The findings suggest that the impact of early‐life OM can be severe enough to interfere with children's development and may result in detrimental long‐term anxiety, attention, and developmental problems that may require professional allied health‐care interventions (e.g., psychologists, speech‐language pathologists, etc.).

Previous investigations of behavioural outcomes in the same cohort revealed that behavioural problems were present in the rOM group at ages 5 and 8, which indicates a continuation of problems since early childhood.^8^ Conjointly, these findings suggest that the cumulative history of OM since early childhood is likely a significant contributor to the behavioural problems observed at 10 years of age, which has also significantly impacted children's cognitive and educational outcomes. The mechanism through which an early history of OM can lead to later behavioural problems is poorly understood and is likely influenced by various environmental and genetic factors not assessed in this study.^9^ However, behavioural problems in children may manifest due to various OM‐related sequelae, such as reduced communication due to hearing loss,^4^ reduced quality of life,^13^ school absence due to illness,^14^ as well as chronic pain and discomfort.

Children in the rOM group were more likely to have attention and hyperactivity problems as measured by the SDQ compared to the reference group. This finding aligns with other studies that revealed an increased likelihood for children and adolescents with early‐life recurrent and/or persistent OM to be inattentive and hyperactive.^3^, ^4^, ^6^, ^15^ In this study, the severity of these issues was further highlighted by the increased rates of diagnoses of attentional problems, indicating that the attention difficulties experienced by children with a history of recurrent OM may have severely impacted their development and academic achievement to the point of warranting professional intervention. Notably, this study is limited by the lack of information regarding the nature and degree of these diagnoses. However, attempts to explain any association between OM and attention problems may lie in the items of the attention‐hyperactivity scale of the SDQ. These items describe characteristics that involve restlessness and a reduced attention span. This is similar to symptoms typically seen in children diagnosed with attention deficit hyperactivity disorder (ADHD) and may also overlap with symptoms seen in children with auditory processing difficulties (APD).^16^ To this connection, several studies have suggested a potential link between early‐life recurrent and/or persistent OM with later ADHD^17^, ^18^, ^19^ and APD.^20^, ^21^, ^22^, ^23^ In the current study, however, associations between OM and ADHD could not be supported as the predictive validity of the SDQ to screen for ADHD is controversial, with different studies showing different degrees of sensitivity.^24^ Nevertheless, our results add to the growing body of literature that links a history of OM with attention and hyperactivity problems. This emphasises the need for future studies to implement more rigorous, validated assessments of attention to differentiate between different types of attentional difficulties, if any, and to determine whether attention problems observed in this population reflect the involvement of other cognitive processes linked to ADHD and APD. This will potentially allow for more appropriate management strategies and maximise efforts to address their educational needs.

### Strengths and limitations

One strength of the current study is the use of data from a population‐based sample, which reduces selection bias. We were able to control for a range of relevant confounding variables to allow for a more reliable investigation of any associations between OM and behavioural problems. Investigations of diagnoses of mental health and developmental problems, as opposed to the sole reliance on behavioural questionnaires, further strengthened our findings and highlighted the level of impact that recurrent OM may pose on children and their development. However, the lack of information regarding the timing and magnitude of the diagnosed problems limited our understanding of the nature of these associations. A further limitation is the use of parental reports to document a history of recurrent OM, which may be subject to recall bias and limit the ability to explore further associations between frequency, type and severity of OM on the measured outcomes. Further, the cross‐sectional nature of this study did not allow to control for additional known factors that may contribute to behavioural problems, including the degree and type of hearing loss, in addition to other factors that may explain any associations reported in this study.

## Conclusion

The present study showed an association between early‐life recurrent OM and poorer behavioural scores at the age of 10 years. Additionally, those children were more likely to have anxiety, attention, learning, and language problems as diagnosed by health professionals. These findings expand on the current knowledge regarding this association and recommend early management and frequent monitoring of OM and developmental outcomes of the affected children to mitigate any potential mental health and developmental sequelae that may be associated with OM. This may be accomplished by incorporating screening tools in clinical settings to allow for early intervention through individualised care plans, potentially reducing the impact of these problems on children in the long term.
